# Twenty-five-year changing pattern of gonococcal antimicrobial susceptibility in Shanghai: surveillance and its impact on treatment guidelines

**DOI:** 10.1186/s12879-014-0731-9

**Published:** 2014-12-30

**Authors:** Wei-Ming Gu, Yue Chen, Yang Yang, Lei Wu, Wei-Zhong Hu, Yue-Lan Jin

**Affiliations:** Shanghai Skin Disease Hospital, Shanghai, China; Department of Epidemiology and Community Medicine, Faculty of Medicine, University of Ottawa, Ottawa, Canada

**Keywords:** Neisseria gonorrhoeae, Antimicrobial susceptibility, Multiple drug resistance, Ceftriaxone, Spectinomycin

## Abstract

**Background:**

Antimicrobial resistance of *Neisseria gonorrhoeae* is a serious health problem in China. Gonococcal antimicrobial susceptibility has been monitored in Shanghai since 1988. In this study, we examined the changing pattern of gonococcal antimicrobial susceptibility based on data from *N. gonorrhoeae* isolates collected over the past 25 years.

**Methods:**

Approximately 100–200 isolates each year (1988–2013) were tested for their susceptibility to penicillin (PEN), tetracycline (TET), ciprofloxacin (CIP), ceftriaxone (CRO) and spectinomycin (SPT), using the agar dilution method. Plasmid-mediated *N. gonorrhoeae* antimicrobial resistance, comprising penicillinase-producing *N. gonorrhoeae* (presumed PPNG) and high-level tetracycline resistance *N. gonorrhoeae* (presumed TRNG), were also determined. Breakpoints for susceptibilities followed those described by the Clinical and Laboratory Standard Institute and the European Committee on Antimicrobial Susceptibility Testing.

**Results:**

A high proportion of isolates were resistant to PEN, TET and CIP, ranging from less than 20% at the beginning of the survey, increasing in the late 1990s and reaching over 90% in recent years. The proportion of isolates exhibiting plasmid-mediated resistance exceeded 38% for presumed PPNG and 20% for presumed TRNG in recent years. The proportion of CRO nonsusceptible isolates (MIC ≥ 0.125 mg/L) ranged from 7% to 13% in most of the study years. Almost all isolates were susceptible to SPT. The SPT MIC_90_ was 16–32 mg/L for 2008–2013. The proportion of CRO nonsusceptible-associated multiple-drug-resistant (MDR) isolates was over 5% in most of the study years.

**Conclusions:**

*N. gonorrhoeae* isolates in Shanghai were resistant to PEN, TET and CIP. Furthermore, CRO nonsusceptible and MDR isolates were prevalent. *N. gonorrhoeae* isolates were also found to be susceptible to SPT. It is recommended that the CRO dose be increased from currently recommended 250 mg to 500 mg and that SPT be an alternative in treating urogenital gonorrhea. Our findings highlight the importance of both regional and national surveillance programs for the prompt modification of treatment guidelines, vital in responding to the changing pattern of gonococcal antimicrobial susceptibility.

**Electronic supplementary material:**

The online version of this article (doi:10.1186/s12879-014-0731-9) contains supplementary material, which is available to authorized users.

## Background

*Neisseria gonorrhoeae*, a Gram-negative bacterium, is responsible for the sexually transmitted disease gonorrhea. In 2008, over 106 million new cases of *N. gonorrhoeae* were reported worldwide [1]. In China, infectious disease surveillance reports more than 100,000 new gonorrhea cases each year. In 2012, there were 95,263 gonorrhea cases reported in China with an incidence rate of 7.07 per 100,000 population. Gonorrhea ranked sixth in the most frequently reported infectious diseases in China [2]. There are currently no vaccines available for *N. gonorrhoeae* [3]. Antimicrobial treatment is essential to control this infectious disease. However, antimicrobial resistance (AMR) in *N. gonorrhoeae* is emerging as a significant public health problem worldwide [4],[5]. In most areas of the world, *N. gonorrhoeae* has developed resistance to sulfonamides, penicillins, tetracyclines, macrolides and fluoroquinolones [6]. Decreased susceptibility, sporadic resistant isolates and clinical treatment failure to oral or parental third-generation cephalosporins have also been observed [7]-[10]. Moreover, extensive/multiple-drug-resistant (MDR) *N. gonorrhoeae* isolates are commonly observed in various areas of the world [8],[10].

In China, the burden of gonococcal penicillin (PEN) and tetracycline (TET) resistance reached high levels [11]-[13]. In response, fluoroquinolones were introduced as a treatment option. Since 1995, orally administered ciprofloxacin (CIP) has been recommended for the treatment of gonorrhea in China. However, as a result, the proportion of CIP resistance drastically increased from 15% in 1996 to nearly 100% in the mid-2000s in China [11]-[13]. The use of CIP for the treatment of gonorrhea has since been terminated in China [14],[15]. Since 2007, ceftriaxone (CRO) has been recommended as the first line drug for the treatment of gonorrhea in China [14]. However, in Shanghai CRO has been used since 2000, and the presence and spread of gonococcal isolates with nonsusceptibility to CRO (minimal inhibitory concentration (MIC) = 0.125–0.25 mg/L) was reported in 11.9% of isolates in 2005 and 19.7% of isolates in this city in 2008 [11],[12].

In this study, we used the data of the Shanghai Gonococcal Antimicrobial Susceptibility Surveillance Program from *N. gonorrhoeae* isolates collected over the past 25 years to analyze trends in AMR development in Shanghai. The AMR data were aligned with the timeline of treatment guidelines for gonorrhea in China. We found that *N. gonorrhoeae* isolates in Shanghai were resistant to PEN, TET and CIP, which were historically used for the treatment of gonorrhea in this region. Plasmid-mediated *N. gonorrhoeae* antimicrobial resistance has been prevalent in the past decades. High proportions of *N. gonorrhoeae* isolates are nonsusceptible to the currently recommended treatment regime of CRO (MIC ≥ 0.125). In Shanghai, MDR associated with the CRO nonsusceptible phenotype was prevalent in *N. gonorrhoeae* isolates. Our report indicates the usefulness of regional surveillance data in the modification of treatment guidelines and provides insights that will be important in future strategic planning for the treatment of gonorrhea.

## Methods

### Collection of *N. gonorrhoeae*isolates

The Shanghai Gonococcal Antimicrobial Susceptibility Surveillance Program was established in 1988 and has been led by the Shanghai Skin Disease Hospital (SSDH), Shanghai, China. The number of gonorrhea cases reported by the SSDH was approximately 14–16% of the reported cases from the entire Shanghai city over the years [16]. The number of gonococcal isolates tested for antimicrobial susceptibility accounted for about 10% of the SSDH reported cases or 2.1% of reported cases in Shanghai in 2012. All of the surveyed *N. gonorrhoeae* isolates were from the Sexually Transmitted Infection Clinic of the SSDH.

Each year in the SSDH, approximately 100–200 *N. gonorrhoeae* isolates were collected from individual patients for antimicrobial susceptibility testing. The first 10–20 *N. gonorrhoeae* isolates each month were collected from patients with culture positive gonorrhea [12]. During the period from 1988 to 2000, *N. gonorrhoeae* isolates were collected from both male and female patients, and during the period from 2001 to 2013, *N. gonorrhoeae* isolates were only collected from male patients. *N. gonorrhoeae* was isolated and cultured as previously described [12].

Gonococcal isolates were collected as part of standard patient care, and ethics approval was not required for their use.

### Determination of the minimal inhibitory concentrations (MIC)

The antimicrobial susceptibility of *N. gonorrhoeae* isolates was determined using the agar dilution method [17] at the Laboratory Diagnostic Centre of the SSDH. WHO reference strains A, B, C, D and E were used in susceptibility tests [12]. Five antimicrobial agents were tested against *N. gonorrhoeae* isolates: PEN (0.002–32.0 mg/L), TET (0.002–32.0 mg/L), CIP (0.002–32.0 mg/L), spectinomycin (SPT; 2.0–128.0 mg/L) and CRO (0.002–2.0 mg/L). Antimicrobial agents were provided by the China GASP at the National Center for Sexually Transmitted Disease Control, China (1988–2002) or purchased from Sigma-Aldrich (Distributor: the Shanghai ANPEL Scientific Instrument Co. Ltd., Shanghai, China). The MIC testing was performed on GC agar base supplemented with 1% IsoVitalex as previously described [12]. Each MIC determination was performed in duplicate. Gonococcal isolates with inconsistent CRO MICs between the duplicates were re-tested.

### Determination of plasmid-mediated resistance in *N. gonorrhoeae*isolates

Beta-lactamase producing *N. gonorrhoeae* (PPNG) isolates were determined using the chromogenic cephalosporin test before 2003 and the nitrocefin test in 2003–2013 (Oxoid; distributed by GuangZhou LOSO Science Ltd.) [12]. All *N. gonorrhoeae* isolates were tested for β-lactamase production. Plasmid-mediated tetracycline-resistant *N. gonorrhoeae* (TRNG) isolates were defined as those having a TET MIC of ≥ 16.0 mg/L. PP/TRNG isolates were defined as those isolates that were β-lactamase positive and had a TET MIC of ≥ 16 mg/L. Therefore, the characterizations for presumed PPNG and TRNG were solely based on the detection of β-lactamase and the TET MIC, respectively.

### Criteria for resistant phenotypes of *N. gonorrhoeae*

Breakpoints for the susceptibilities to PEN, TET, CIP and SPT were those described by the Clinical Laboratory Standards Institute (CLSI) [17]. The classifications of resistance phenotypes included PEN resistance (MIC ≥ 2.0 mg/L), TET resistance (MIC ≥ 2.0 mg/L), CIP resistance (MIC ≥ 1.0 mg/L) and SPT resistance (MIC ≥ 128.0 mg/L). Chromosomally-mediated resistant *N. gonorrhoeae* (CMRNG) isolates exhibited a PEN MIC of ≥ 2.0 mg/L, a TET MIC of ≥ 2.0 mg/L and were not classified as PPNG or TRNG. For CRO susceptibility, the European Committee on Antimicrobial Susceptibility Testing (EUCAST) breakpoint was followed (nonsusceptible, MIC ≥ 0.125 mg/L) [18]. In this study, the susceptibilities to CRO in *N. gonorrhoeae* were further categorized as susceptible (MIC ≤ 0.03 mg/L), reduced susceptibility (MIC = 0.06) and nonsusceptible (MIC ≥ 0.125 mg/L) [11]. The MDR phenotypes of *N. gonorrhoeae* were defined as various combinations of the above resistant phenotypes (Table 1).

**Table 1:** **Criteria for the phenotypes of multiple drug resistance in** ***N. gonorrhoeae***

Resistant phenotype	PEN	TET	CIP	CRO
CMRNG	≥2	≥2	na	na
PEN-TET	≥2	≥2	na	na
PEN-CIP	≥2	na	≥1	na
PEN-CRO	≥2	na	na	≥0.125
TET-CIP	na	≥2	≥1	na
TET-CRO	na	≥2	na	≥0.125
CIP-CRO	na	na	≥1	≥0.125
PEN-TET-CIP	≥2	≥2	≥1	na
PEN-TET-CRO	≥2	≥2	na	≥0.125
PEN-CIP-CRO	≥2	na	≥1	≥0.125
TET-CIP-CRO	na	≥2	≥1	≥0.125
PEN-TET-CIP-CRO	≥2	≥2	≥1	≥0.125

*CMRNG*: chromosomally-mediated resistant *N. gonorrhoeae*, non-penicillinase-producing *N. gonorrhoeae* (Non-PPNG) and tetracycline MIC <16 mg/L.

*PEN*: penicillin; *TET*: tetracycline; *CIP*: ciprofloxacin; *CRO*: ceftriaxone (nonsusceptible); na: not applicable; MICs are presented as mg/L; Spectinomycin (SPT) was not listed.

## Results

### *N. gonorrhoeae*has become less susceptible to CRO

During the period from 1995 to 2013, 3153 *N. gonorrhoeae* isolates were examined for their susceptibility to CRO (Table 2). Overall, 71.90% isolates were susceptible to CRO (MIC ≤ 0.03 mg/L); 20.30% had a reduced susceptibility phenotype (MIC = 0.06 mg/L) and 7.80% exhibited a CRO nonsusceptible phenotype (MIC ≥ 0.125 mg/L). The highest CRO MIC value for the *N. gonorrhoeae* isolates was 0.25 mg/L, which accounted for 0.92% of the isolates tested (29/3153).

**Table 2:** **Susceptibility of** ***N. gonorrhoeae*** **isolates to ceftriaxone in Shanghai**

Year	N	Reduced*		Nonsusceptible**		MIC _50_	MIC _90_	MIC range
		n	%	n	%	(mg/L)	(mg/L)	(mg/L)
1995	117	0	0.00	0	0.00	0.008	0.015	0.008-0.03
1996	206	2	0.97	2	0.97	0.008	0.03	0.002-0.25
1997	208	7	3.37	2	0.96	0.008	0.03	0.002-0.25
1998	180	29	16.11	14	7.78	0.03	0.06	0.004-0.25
1999	208	27	12.98	12	5.77	0.015	0.06	0.002-0.25
2000	225	47	20.89	15	6.67	0.03	0.06	0.004-0.25
2001	230	39	16.96	16	6.96	0.03	0.06	0.004-0.25
2002	210	30	14.29	20	9.52	0.03	0.06	0.004-0.25
2003	302	57	18.87	34	11.26	0.03	0.125	0.004-0.25
2004	166	53	31.93	20	12.05	0.03	0.125	0.004-0.25
2005	83	26	31.33	5	6.02	0.03	0.06	0.004-0.125
2006	128	51	39.84	18	14.06	0.06	0.125	0.008-0.25
2007	200	64	32.00	10	5.00	0.03	0.06	0.004-0.25
2008	71	25	35.21	14	19.72	0.06	0.125	0.008-0.125
2009	157	36	22.93	18	11.46	0.03	0.125	0.008-0.125
2010	119	34	28.57	9	7.56	0.03	0.06	0.008-0.125
2011	135	55	40.74	14	10.37	0.06	0.125	0.008-0.125
2012	99	14	14.14	7	7.07	0.03	0.06	0.004-0.125
2013	109	43	39.45	15	13.76	0.06	0.125	0.004-0.25
Total	3153	639	20.30	245	7.80			

*: CRO MIC = 0.06 mg/L; **: CRO MIC ≥ 0.125 mg/L.

The proportions of CRO non-susceptible isolates were over or equal to 5% in all years since 1998, and were around 7%-13% in the last 3 years (2011 to 2013). The proportion of isolates showing reduced susceptibility to CRO ranged from 12% to 40% from 1998 to 2013. From 1998 to 2013, the MIC_50_ values for CRO were between 0.015 mg/L and 0.06 mg/L, and the MIC_90_ values were 0.06–0.125 mg/L (Table 2).

### *N. gonorrhoeae*is resistant to CIP, PEN and TET

During the period from 1996 to 2013, 3036 *N. gonorrhoeae* isolates were examined for susceptibility to CIP. Overall, 85.30% isolates were resistant to CIP. CIP resistant *N. gonorrhoeae* accounted for 16.50% of the *N. gonorrhoeae* isolates in 1996, compared with over 85% in 1999. CIP resistance among the isolates ranged from 88% to 100% in the period between 2000 and 2013 (Additional file 1: Figure S1). A total of 3663 *N. gonorrhoeae* isolates were examined for their susceptibility to PEN. Overall, 62.00% isolates were resistant to PEN. Proportions of PEN resistance increased from 20–40% before 1998, to 70–90% during the period from 2000 to 2013 (Additional file 1: Figure S1). During the period from 1995 to 2013, 3153 *N. gonorrhoeae* isolates were examined for their susceptibility to TET. Only one concentration of TET (16 mg/L) was tested during 1998–2003 (n = 1355), whereas the remaining 1798 *N. gonorrhoeae* isolates were tested for the full range of TET concentrations. For the 1798 isolates tested during 1995–1997 and 2004–2013, 50.61% (910/1798) were resistant to TET. The proportion of TET resistant isolates increased from 16% (1995–1997) to over 40% (2004–2012), and then further increased to 82.57% in 2013 (Additional file 1: Figure S1).

### Susceptibility of *N. gonorrhoeae*isolates to SPT

From 1995 to 2013, 3153 *N. gonorrhoeae* isolates were examined for their susceptibility to SPT. Only two resistant isolates (0.06%, MIC ≥ 128 mg/L) were identified, one in 1999 and the other in 2002. Overall, isolates with intermediate resistance (MIC = 64 mg/L) accounted for 6.76%, and the related proportion of susceptible isolates was 93.18%. During the period from 2009 to 2013, the proportion of isolates with an SPT MIC ≥ 64 mg/L was less than 5% (4.46% in 2009, 0% in 2010, 2.22% in 2011 and 0% in 2012 and 2013), with the SPT MIC_50_ ranging between 4 mg/L and 32 mg/L during this period. The majority of SPT MIC_90_ values were 16–32 mg/L, and the values of MIC_90_ in 2003 and 2007 were 64 mg/L (data not shown).

### Plasmid-mediated and chromosomally-mediated resistant *N. gonorrhoeae*

In 1995, only 0.85% of isolates exhibited a presumed PPNG phenotype. Around 5% of isolates were presumed PPNG from 1996 to 1998. The proportion of presumed PPNG isolates increased rapidly from 13% in 1999 to 37% in 2000 and ranged from 37% to 57% during the period from 2001 to 2013 (Additional file 1: Figure S2). The proportion of presumed TRNG isolates was 0–1% for the period from 1995 to 1998, and increased to 7.69% and 6.67% for 1999 and 2000, respectively. During 2001–2005, the proportion of presumed TRNG was around 17% - 23%. This proportion again increased from 30% (2006) to 56% (2008), and remained above 30%, from 2009 to 2013 (Additional file 1: Figure S2). There were no PP/TRNG isolates detected during the first 4 years of surveillance (1995–1998). The proportion of presumed PP/TRNG isolates was 0.48% and 1.78% for 1999 and 2000, respectively. This proportion increased rapidly to 12–14% during the period from 2001 to 2005 and remained above 15% between 2006 and 2013, peaking at 33.8% in 2008 (Additional file 1: Figure S2).

The proportion of chromosomally-mediated resistance to PEN and TET among the 1569 CMRNG isolates from 2003 to 2013 ranged from 4.23% to 29.14%. In recent years, the proportion of CMRNG isolates detected has been 15.56% (2011), 6.06% (2012) and 23.85% (2013).

### Multiple drug resistance (MDR) in *N. gonorrhoeae*

An MDR phenotype for *N. gonorrhoeae* was defined as resistance to two or more antimicrobial agents (Table 1). Double-resistant phenotypes accounted for 39–68% (PEN-TET), 61–92% (PEN-CIP) and 43–81% (TET-CIP) of *N. gonorrhoeae* isolates, and the triple-resistant phenotype (PEN-TET-CIP) accounted for 38–61% of *N. gonorrhoeae* isolates during the period from 2003 to 2013 (data not shown).

Between 2001 and 2013, the proportion of the CRO nonsusceptible phenotype combined with PEN, TET or CIP resistance fluctuated from 4.5% to 19.72% (PEN-CRO), 2.41% to 12.68% (TET-CRO) or 0% to 19.72% (CIP-CRO) (Table 3). CRO-associated triple-resistant phenotypes accounted for 2.41–12.68% (PEN-TET-CRO), 2.41–12.68% (TET-CIP-CRO) and 2.17–19.72% (PEN-CIP-CRO) of *N. gonorrhoeae* isolates (Table 3), and the proportion of quadruple resistant phenotypes (PEN-TET-CIP-CRO) accounted for 2.41–12.68% of *N. gonorrhoeae* isolates (Table 3).

**Table 3:** **MDR associated with the CRO nonsusceptible phenotype in** ***N. gonorrhoeae*** **isolates in Shanghai**

Year	N	PEN-CRO	TET-CRO	CIP-CRO	PEN-TET-CRO	TET-CIP-CRO	PEN-CIP-CRO	PEN-TET -CIP-CRO
2001	230	6.52%	-	6.52%	-	-	2.17%	-
2002	210	7.62%	-	9.52%	-	-	3.81%	-
2003	302	9.60%	9.93%	10.26%	8.94%	8.94%	8.61%	7.95%
2004	166	12.05%	7.83%	12.05%	7.83%	7.83%	12.05%	7.83%
2005	83	6.02%	2.41%	0.00%	2.41%	2.41%	6.02%	2.41%
2006	128	12.50%	7.03%	13.28%	6.25%	7.03%	12.50%	6.25%
2007	200	4.50%	3.00%	5.00%	2.50%	3.00%	4.50%	2.50%
2008	71	19.72%	12.68%	19.72%	12.68%	12.68%	19.72%	12.68%
2009	157	11.64%	7.64%	11.46%	7.64%	7.64%	11.46%	7.64%
2010	119	7.56%	7.56%	7.56%	7.56%	7.56%	7.56%	7.56%
2011	135	10.37%	8.15%	10.37%	8.15%	8.15%	10.37%	8.15%
2012	99	6.06%	5.05%	7.07%	5.05%	5.05%	6.06%	5.05%
2013	109	11.93%	10.09%	13.76%	9.17%	10.09%	11.93%	9.17%

## Discussion

In China, antibiotic resistance of *N. gonorrhoeae* has become an increasing problem over the past few decades in the treatment of gonorrhea infection. Since the mid-1990s, a high proportion of *N. gonorrhoeae* isolates sampled in Shanghai were resistant to PEN, TET and CIP. Plasmid-mediated resistance has been highly prevalent since the early 2000s. The proportion of *N. gonorrhoeae* isolates exhibiting a CRO nonsusceptible phenotype (MIC ≥ 0.125 mg/L) has been greater than 5% since 1998. In 2013, 13.76% of isolates exhibited a CRO MIC of ≥ 0.125 mg/L, suggesting that the effectiveness of the currently recommended treatment regime for gonorrhea is diminishing. MDR phenotypes associated with PEN, TET and CIP resistance were prevalent. *N. gonorrhoeae* isolates exhibiting a CRO nonsusceptible phenotype associated MDR were also found to be prevalent in recent years. In Shanghai, *N. gonorrhoeae* isolates were found to be susceptible to SPT.

The CRO MIC of *N. gonorrhoeae* isolates has been extensively monitored. In the United States, the proportion of isolates with elevated CRO MICs (≥0.125 mg/L) ranged from 0.1% to 0.4% between 2006 and 2012 [19]. In Canada, 7.3% of *N. gonorrhoeae* isolates showed CRO MICs ≥ 0.125 mg/L in 2010 [20]. In England and Wales during 2007–2011, 0–0.4% of *N. gonorrhoeae* isolates showed CRO MICs ≥ 0.125 mg/L [4]. In Australia, no isolates of *N. gonorrhoeae* with CRO MIC values ≥ 0.125 mg/L were reported in 2011 [21],[22]. In Latin America and the Caribbean countries, 0.4% of *N. gonorrhoeae* isolates had CRO MICs ≥ 0.125 mg/L between 2007 and 2011 [23].

In the present study, we found that the proportion of *N. gonorrhoeae* isolates with CRO MICs ≥ 0.125 mg/L in Shanghai (1996–2013) greatly varied from year to year, ranging from 0% (1995) to 19.72% in 2008, and 13.76% in 2013. Our findings were consistent with those of a survey of CRO susceptibility in Nanjing, China [24],[25]. The proportion of CRO less susceptible *N. gonorrhoeae* isolates (CRO MIC ≥ 0.06 mg/L) was 17.86–50.5% during the period from 1999 to 2006 in Nanjing [24]. Chen *et al.* reported that the proportion of isolates with a CRO MIC = 0.125 mg/L was 39.4% in 2007 and 11.3% in 2012 [25]. The proportion of CRO nonsusceptible isolates (MIC ≥ 0.125 mg/L) ranged from 22% to over 48% in Nanning in 2000–2012 [26], much higher than that detected in Shanghai. Regional differences in gonococcal AMR within a country have also been demonstrated in other countries. In Canada, isolates of *N. gonorrhoeae* from the province of Saskatchewan retained a high prevalence of susceptibility to antibiotics, including CIP and PEN and CRO. Unlike reports of reduced susceptibility to third-generation cephalosporins (CRO or cefixime) from other regions of Canada and the rest of the world, all isolates collected during 2003–2008 from Saskatchewan were fully susceptible to CRO and cefixime [27]. Approximately 7% of the isolates (n = 23) showed a CRO MIC of 0.03–0.06 mg/L, and 93% of the isolates (n = 297) showed a CRO MIC of < 0.03 mg/L [27]. In Canada between 2000 and 2009 (n = 40,875), the proportions of PEN, TET or CIP resistant isolates were 11.8%, 21.3% and 13.2%, respectively; and the CRO MIC had shifted from 0.016 mg/L in 2000 to 0.063 mg/L in 2009 [28]. In British Columbia (2006–2011), 13% of isolates (227/1837) showed a CRO MIC of 0.064 mg/L and 1% of isolates (27/1837) showed a CRO MIC of 0.125 mg/L [29]. Indeed, cefixime treatment failure was found in 3.09% of the investigated cases (9/291) in Toronto between 2010 and 2011 [9]. Taking all of these data into account, it is clear that regional monitoring of *N. gonorrhoeae* AMR is as important as national surveillance programs.

Currently, the recommended therapy for gonorrhea is a single dose of oral or parental third-generation cephalosporin. The doses of CRO used for the treatment of uncomplicated gonorrhea vary among countries; typically 250–500 mg per dose is injected intramuscularly, but in Japan 1 g is administered intravascularly [14],[23],[30]-[34]. A landmark study suggested that increasing the dose of CRO is associated with a decreased proportion of CRO nonsusceptible *N. gonorrhoeae* isolates in the UK [4]. However, this decrease may not be sustainable [21]. We observed that the proportion of CRO nonsusceptible isolates transiently increased from 5% in 2007 when CRO became a recommended drug in China, to over 19% in 2008, 11% in 2009 and 13% in 2013. In Shanghai, CRO at a single low dose (250 mg) has been used to treat uncomplicated gonorrhea since 2000, and the proportion of CRO nonsusceptible *N. gonorrhoeae* isolates remains fairly stable. Based on our findings and reports from others in China [24]-[26], CRO remains an effective antimicrobial for the treatment of uncomplicated gonorrhea. Since CRO nonsusceptible *N. gonorrhoeae* isolates are prevalent in China, a higher dose of CRO (500 mg) is recommended, at least in some areas such as Shanghai, Nanjing and Nanning [24]-[26].

Detailed data on trends of MDR of *N. gonorrhoeae* in China are limited. However, with the consideration of the high resistant proportions of gonococcal isolates to PEN, TET, and CIP, non-susceptible CRO-associated MDR should not be ignored in China. We observed that over 5% of isolates were non-susceptible CRO-associated MDR in Shanghai in recent years.

One of the major purposes of antimicrobial susceptibility surveillance is to provide information that may indicate the need for modification of treatment guidelines. The World Health Organization recommended that when the prevalence of resistant gonococci to an antimicrobial agent rises to 5%, the antimicrobial should no longer be used [35]. In China, PEN had been the treatment of choice for gonorrhea until quinolones were introduced in 1993, PEN was removed from the treatment regime in 2000 [14],[15]. Before 2000, the recommended dose of PEN continually increased because of a progressive decline in susceptibility. Penicillin was removed from the treatment regime 10 years after it was discontinued in 1985 in most regions of the world, simply because of the availability of replacing antibiotics. In 1993, oral fluoroquinolones (CIP and ofloxacin) were recommended for gonorrhea treatment, and at that time CIP resistance was already 15% in China [12],[13]. Four years after (1997), CIP resistance reached over 80% (100% in 2000), while the drug remained in the treatment regime until 2007 [14].

Recently potential novel treatment strategies have been explored [6],[7]. A strategy of combining older drugs was discussed [36]-[38]. However, there is no evidence that this combination of older drugs produces synergistic effects. Considering the problems of MDR, this strategy should be used with caution in China. A high prevalence of MDR to almost all currently available drugs in Shanghai limits options for combined therapy. New antibiotics are needed but are largely unavailable [38]. It is therefore worth considering and evaluating SPT as an alternative first line drug for the treatment of uncomplicated urogenital gonorrhea in China. However, SPT is not suitable for the treatment of pharyngeal gonorrhea even though the prevalence of resistance is low in most areas of the world [39]. The MICs of SPT need to be closely monitored, and any signs of increased SPT MIC warrant a thorough investigation and close attention by the policy makers.

The current study has a number of limitations. 1) All of the *N. gonorrhoeae* isolates tested in this study were collected from the Sexually Transmitted Infection Clinic of the SSDH, and only a small number of isolates were tested, accounting for 2.1% of the total reported cases in 2012 in Shanghai. 2) A lack of demographic and clinical data prevented any comparisons of *N. gonorrhoeae* antimicrobial resistance profiles with patient characteristics; 3) Molecular subtyping of the isolates was not performed. This would provide insight into whether there is clonal spread of resistant isolates and should be the focus of future studies. 4) The agar dilution MIC testing method has an intrinsic error rate of plus/minus one dilution. While using a breakpoint of 0.125 mg/L for CRO nonsusceptible isolates, some isolates may actually have MICs of 0.06 mg/L.

## Conclusions

In summary, antimicrobial resistance of *N. gonorrhoeae* to PEN, TET and CIP is highly prevalent in Shanghai. A high proportion of *N. gonorrhoeae* isolates were found to be CRO nonsusceptible (MIC ≥ 0.125 mg/L). Plasmid-mediated resistance and MDR are also prevalent in China. *N. gonorrhoeae* isolates are susceptible to SPT and this antibiotic needs to be further evaluated as a treatment option for urogenital gonococcal infections. Our findings suggest that the CRO dose should be increased to 500 mg in areas with a high prevalence of CRO nonsusceptible isolates. We propose that combination therapy using older antibiotics should be used with caution unless drug synergistic effects can be confirmed. Finally, both national and regional antimicrobial susceptibility surveillance programs are important to inform policy makers in a timely manner on the appropriate modifications of treatment guidelines.

## Additional file

## Electronic supplementary material

Additional file 1: Figure S1.: Resistant proportions of *N. gonorrhoeae* isolates to CIP, PEN and TET. Closed diamond solid line: CIP resistant; Open square dashed line: PEN resistant; Closed triangle solid line: TET resistant. Surveillance of PEN susceptibility started in 1988, and data to PEN in 1990, 1991 and 1994 were not available. Surveillance of CIP and TET susceptibility started in 1995 (TET) and 1996 (CIP). During 1998–2002, TET was examined only at a concentration of 16 mg/L, and proportions of resistance were unavailable. N: number of isolates tested. R: resistant. **Figure S2.** Proportions of plasmid-mediated resistance in *N. gonorrhoeae*. Closed diamond solid line: Penicillinase-producing *N. gonorrhoeae* (presumed PPNG); Open square dashed line: High level TET resistance *N. gonorrhoeae* (presumed TRNG); Closed triangle dashed line: presumed PP/TRNG. N: number of isolates tested. (DOCX 85 KB)

## Abbreviations

AMR: Antimicrobial resistance
CLSI: Clinical Laboratory Standard Institute
CIP: Ciprofloxacin
CMRNG: Chromosomally-mediated resistant *N. gonorrhoeae*
CRO: Ceftriaxone
EUCAST: European Committee on Antimicrobial Susceptibility Testing
MDR: Multiple drug resistance
MIC: Minimal inhibitory concentration
PEN: Penicillin
PPNG: Penicillinase-producing *N. gonorrhoeae*
SPT: Spectinomycin
SSDH: Shanghai Skin Disease Hospital
TET: Tetracycline
TRNG: High-level tetracycline-resistant *N. gonorrhoeae*

## References

[1] World Health Organization (WHO) (2012). Global incidence and prevalence of selected curable sexually transmitted infections - 2008.

[2] Qiu X, Jiang N, Gong X (2013). [Reports on syphilis and gonorrhea in China in 2012]. Bulletin Sex Transm Dis.

[3] Jerse AE, Deal CD (2013). Vaccine research for gonococcal infections: where are we?. Sex Transm Infect.

[4] Ison CA, Town K, Obi C, Chisholm S, Hughes G, Livermore DM, Lowndes CM (2013). GRASP Collaborative group: Decreased susceptibility to cephalosporins among gonococci: data from the Gonococcal Resistance to Antimicrobials Surveillance Programme (GRASP) in England and Wales, 2007–2011. Lancet Infect Dis.

[5] Ndowa FJ, Ison CA, Cole MJ, Lusti-Narasimhan M (2013). Gonococcal antimicrobial resistance: challenges for public health control. Sex Transm Infect.

[6] Unemo M, Shafer WM (2014). Antimicrobial resistance in *Neisseria gonorrhoeae* in the 21^st^century: past, evolution, and future. Clin Microbiol Rev.

[7] Ison CA, Deal C, Unemo M (2013). Current and future treatment options for gonorrhoea. Sex Transm Infect.

[8] Unemo M, Nicholas RA (2012). Emergence of multidrug-resistant, extensively drug-resistant and untreatable gonorrhea. Future Microbiol.

[9] Allen VG, Mitterni L, Seah C, Rebbapragada A, Martin IE, Lee C, Siebert H, Towns L, Melano RG, Low DE (2013). *Neisseria gonorrhoeae*treatment failure and susceptibility to cefixime in Toronto, Canada. JAMA.

[10] Lewis DA (2014). Global resistance of *Neisseria gonorrhoeae*: when theory becomes reality. Curr Opin Infect Dis.

[11] Liao M, Bell K, Gu WM, Yang Y, Eng NF, Fu W, Wu L, Zhang CG, Chen Y, Jolly AM, Dillon JA (2008). Clusters of circulating *Neisseria gonorrhoeae*strains and association with antimicrobial resistance in Shanghai. J Antimicrob Chemother.

[12] Yang Y, Liao M, Gu WM, Bell K, Wu L, Eng NF, Zhang CG, Chen Y, Jolly AM, Dillon JA (2006). Antimicrobial susceptibility and molecular determinants of quinolone resistance in *Neisseria gonorrhoeae*isolates from Shanghai. J Antimicrob Chemother.

[13] Ye S, Su X, Wang Q, Yin Y, Dai X, Sun H (2002). Surveillance of antibiotic resistance of *Neisseria gonorrhoeae*isolates in China, 1993–1998. Sex Transm Dis.

[14] Wang Q, Zhang G (2007). [Guidelines for diagnosis and treatment of sexually transmitted diseases 2007]. Center for Control of Sexually Transmitted Diseases, Center for Disease Control, China (China CDC).

[15] Ministry of Health China (2000). [Guidelines for the treatment of sexually transmitted infections 2000].

[16] National Centre for STD Control, China CDC: [Bulletin of Sexually Transmitted Diseases], [Available at: http://www.ncstdc.org/list.asp?id=185; 4 May 2014, date last accessed).

[17] Clinical and Laboratory Standard Institute (2014). Performance standards for antimicrobial susceptibility testing: Twenty-fourth informational supplement M100-S24.

[18] European Committee on Antimicrobial Susceptibility Testing (EUCAST): Expert rules 2010 [Available at: http://www.eucast.org/expert_rules. AST to Bacteria Breakpoint Table Version 1.1.; 3 May 2014, date last accessed].

[19] Kirkcaldy RD, Kidd S, Weinstock HS, Papp JR, Bolan GA (2013). Trends in antimicrobial resistance in *Neisseria gonorrhoeae*in the USA: the Gonococcal Isolate Surveillance Project (GISP), January 2006-June 2012. Sex Transm Infect.

[20] Martin I, Sawatzky P, Liu G, Allen V, Lefebvre B, Hoang L, Lovgren M, Haldane D, Caeseele PV, Horsman G, Garceau R, Ratnam S, Wong T, Gilmour M (2013). Antimicrobial susceptibilities and distribution of sequence types of *Neisseria gonorrhoeae*isolates in Canada: 2010. Can J Microbiol.

[21] Lahra MM, Donovan B, Whiley DM (2014). Decreased susceptibility to cephalosporins among gonococci?. Lancet Infect Dis.

[22] Lahra MM (2012). Australian Gonococcal Surveillance Programme: Annual report of the Australian Gonococcal Surveillance Programme, 2011. Commun Dis Intell.

[23] Dillon JA, Trecker MA, Thakur SD (2013). Gonococcal Antimicrobial Surveillance Program Network in Latin America and Caribbean 1990–2011: Two decades of the gonococcal antimicrobial surveillance program in South America and the Caribbean: challenges and opportunities. Sex Transm Infect.

[24] Su X, Jiang F, Ge Q, Dai X, Sun H, Ye S (2007). Surveillance of antibmicrobial susceptibilitites in *Neisseria gonorrhoeae*in Nanjing, China, 1999–2006. Sex Transm Dis.

[25] Chen SC, Yin YP, Dai XQ, Unemo M, Chen XS (2014). Antimicrobial resistance, genetic resistance determinants for ceftriaxone and molecular epidemiology of *Neisseria gonorrhoeae*isolates in Nanjing, China. J Antimicrob Chemother.

[26] Zhu BY, Yu RX, Yin Y, Chen X, Li W, Dai XQ, Liang M, Gan Q, Huang YJ, Wei JP (2014). Surveillance of antimicrobial susceptibilities of *Neisseria gonorrhoeae*in Nanning, China, 2000–2012. Sex Transm Dis.

[27] Thakur SD, Starnino S, Horsman GB, Levett PN, Dillon JR (2014). Unique combined penA/mtrR/porB mutations and NG-MAST strain types associated with ceftriaxone and cefixime MIC increases in a ‘susceptible’ *Neisseria gonorrhoeae*population. J Antimicrob Chemother.

[28] Martin I, Jayaraman G, Wong T, Liu G, Gilmour M (2011). Canadian Public Health Laboratory Network: Trends in antimicrobial resistance in *Neisseria gonorrhoeae*isolated in Canada: 2000–2009. Sex Transm Dis.

[29] Hottes TS, Lester RT, Hoang LM, McKay R, Imperial M, Gilbert M, Patrick D, Wong T, Martin I, Ogilvie G (2013). Cephalosporin and azithromycin susceptibility in Neisseria gonorrhoeae isolates by site of infection, British Columbia, 2006 to 2011. Sex Transm Dis.

[30] Public Health Agency of Canada: *Canadian Guidelines on Sexually Transmitted Infections, 2008 Edition*. Public Health Agency of Canada. Ottawa, ON, Canada; 2008.

[31] Bignell C, Fitzgerald M (2011). UK national guideline for the management of gonorrhea in adults, 2011. Int J STD AIDS.

[32] Sexual Health Society of Victoria (2008). National Management Guidelines for Sexually Transmitted Infections 7th edition.

[33] Centers for Disease Control and Prevention (2012). Update to CDC’s Sexually transmitted diseases treatment guidelines, 2010: oral cephalosporins no longer a recommended treatment for gonococcal infections. Morb Mortal Wkly Rep.

[34] Centers for Disease Control and Prevention (2010). Sexually transmited disease treatment guidelines, 2010. Morb Mortal Wkly Rep.

[35] World Health Organization: *Antimicrobial resistance in Neisseria gonorrhoeae. Available at WHO/CDS/CSR/DRS/2001.3. 2001*. (3 May 2014, date last accessed). http://www.who.int/drugresistance/Antimicrobial_resistance_in_Neisseria_gonorrhoeae.pdf,

[36] Pereira R, Cole MJ, Ison CA (2013). Combination therapy for gonorrhoea: in vitro synergy testing. J Antimicrob Chemother.

[37] Lusti-Narasimhan M, Pessoa-Silva CL, Temmerman M (2013). Moving forward in tackling antimicrobial resistance: WHO actions. Sex Transm Infect.

[38] Ndowa F, Lusti-Narasimhan M, Unemo M (2012). The serious threat of multidrug-resistant and untreatable gonorrhoea: the pressing need for global action to control the spread of antimicrobial resistance, and mitigate the impact on sexual and reproductive health. Sex Transm Infect.

[39] Gil-Setas A, Navascués-Ortega A, Beristain X: Spectinomycin in the treatment of gonorrhoea. Euro Surveill, 2010; 15:pii = 19568. Available at http://www.eurosurveillance.org/ViewArticle.aspx?ArticleId=19568. (3 May 2014, date last accessed). 20483105

