# Supplementary material for: Twenty-five-year changing pattern of gonococcal antimicrobial susceptibility in Shanghai: surveillance and its impact on treatment guidelines
Source: BMC Infect Dis. 2014 Dec 30;14:731. doi: 10.1186/s12879-014-0731-9 (PMC4334756; doi:10.1186/s12879-014-0731-9)
Supplement: Supplementary file 1 — Additional file 1: Figure S1.: Resistant proportions of N. gonorrhoeae isolates to CIP, PEN and TET. Closed diamond solid line: CIP resistant; Open square dashed line: PEN resistant; Closed triangle solid line: TET resistant. Surveillance of PEN susceptibility started in 1988, and data to PEN in 1990, 1991 and 1994 were not available. Surveillance of CIP and TET susceptibility started in 1995 (TET) and 1996 (CIP). During 1998–2002, TET was examined only at a concentration of 16 mg/L, and proportions of resistance were unavailable. N: number of isolates tested. R: resistant. Figure S2. Proportions of plasmid-mediated resistance in N. gonorrhoeae. Closed diamond solid line: Penicillinase-producing N. gonorrhoeae (presumed PPNG); Open square dashed line: High level TET resistance N. gonorrhoeae (presumed TRNG); Closed triangle dashed line: presumed PP/TRNG. N: number of isolates tested. (DOCX 85 KB) [file 12879_2014_731_MOESM1_ESM.docx]

**Supplement**

**Gu et al: Twenty-five-year changing pattern of gonococcal antimicrobial susceptibility in Shanghai: surveillance and its impact on treatment guidelines**

**Fig. 1**

**Proportion of Isolates**

**Year**

**N**

**133 75 100 202 117 206 208 180 208 225 230 210 302 166 83 128 200 71 157 119 135 99 109**

**Fig. 1. Resistant proportions of *N. gonorrhoeae* isolates to CIP, PEN and TET.** Closed diamond solid line: CIP resistant; Open square dashed line: PEN resistant; Closed triangle solid line: TET resistant. Surveillance of PEN susceptibility started in 1988, and data to PEN in 1990, 1991 and 1994 were not available. Surveillance of CIP and TET susceptibility started in 1995 (TET) and 1996 (CIP). During 1998-2002, TET was examined only at a concentration of 16 mg/L, and proportions of resistance were unavailable. N: number of isolates tested. R: resistant

**Fig. 2.**

**Proportion of Isolates**

**Year**

**N**

**117 206 208 180 208 225 230 210 302 166 83 128 200 71 157 119 135 99 109**

**Fig. 2. Proportions of plasmid-mediated resistance in *N. gonorrhoeae*.** Closed diamond solid line: Penicillinase-producing *N. gonorrhoeae* (presumed PPNG); Open square dashed line: High level TET resistance *N. gonorrhoeae* (presumed TRNG); Closed triangle dashed line: presumed PP/TRNG. N: number of isolates tested
